# Evaluation of the Osteogenic Potential of Apigenin via Inducing Autophagy: An In Vitro Study

**DOI:** 10.1155/sci/6446943

**Published:** 2026-02-12

**Authors:** Mozafar Khazaei, Amirmohammad Khodaei, Maryam Bozorgi, Mohammad Rasool Khazaei, Azam Bozorgi

**Affiliations:** ^1^ Fertility and Infertility Research Center, Health Technology Institute, Kermanshah University of Medical Sciences, Kermanshah, Iran, kums.ac.ir; ^2^ Student Research Committee, Kermanshah University of Medical Sciences, Kermanshah, Iran, kums.ac.ir; ^3^ Department of Tissue Engineering, School of Medicine, Kermanshah University of Medical Sciences, Kermanshah, Iran, kums.ac.ir

**Keywords:** apigenin, autophagy, mesenchymal stem cells, osteogenic differentiation

## Abstract

The present study aimed to evaluate the effect of apigenin (Api) on the osteogenic differentiation of adipose tissue‐derived mesenchymal stem cells (ASCs) by stimulating autophagy. ASCs were isolated from fresh adipose tissues using mechanical and enzymatic digestion and characterized using flow cytometry. ASCs were treated with Api (0, 5, 10, 25, and 50 µM) for 48 and 72 h. After determining the optimal doses of Api (5 and 10 µM), ASCs were differentiated into the osteogenic lineage for 7 and 21 days. The expression of osteogenic and autophagy genes and proteins, as well as alkaline phosphatase (*ALP*) activity and calcium deposition, were assessed. About 94% of ASCs expressed CD73, CD90, and CD105, while 99% didn’t express CD34 and CD45. Api treatment increased the expression of *ALP, RUNX2, COL I, osteocalcin (OCN), ATG5, ATG7*, and *LC3A* genes, and *RUNX2*, *OCN*, LC3‐1, and LC3‐II proteins dose‐dependently. *ALP* activity and calcium deposition were significantly higher in Api‐treated groups than in the control. Api increased the osteogenic differentiation of ASCs via inducing autophagy, an effect advantageous for enhancing SC differentiation efficiency.

## 1. Introduction

Bone is a connective tissue with unique mechanical strength, specialized for body movement, shielding vital organs like the brain and heart, and storing calcium and phosphate ions. Bone matrix is composed of organic (collagen and noncollagenous proteins) and inorganic (hydroxyapatite) phases with highly dynamic and frequent remodeling potential, mediated by the coordinated activity of osteoblasts (bone‐forming) and osteoclasts (bone‐resorbing cells) [1]. However, congenital bone malformation, inappropriate lifestyle, traumas and fractures, autoimmune diseases, and medical interventions can reduce bone strength and cause bone defects with delayed regeneration [2].

Considering the variety and complexity of bone disease, a broad range of pharmacological, nonpharmacological, and surgical interventions are applied to ensure bone integrity by regulating osteoblast/osteoclast metabolism and balancing bone formation and resorption [3]. Bone grafting and implantation of autografts, allografts, and xenografts are key clinical interventions for treating bone damage. Despite the beneficial outcomes, this approach is challenging due to limited accessibility, the risk of infections, donor site morbidity, and graft immune rejection [4].

Since the first time Heinrich Boveri and Haecker introduced the definition of stem cell (SC), numerous experimental studies have evaluated the safety and efficacy of SC‐based therapies for alleviating or treating several human diseases. Additionally, clinical evidence has demonstrated the success of SC therapy in treating cancer, cardiovascular diseases, gastrointestinal disorders, and arthritis [5]. MSCs are multipotent SCs found in postnatal and adult tissues, including umbilical cord tissue, blood, bone marrow, and adipose tissue. Although a variety of SC sources have been proposed for clinical applications, mesenchymal SCs (MSCs) outperform others due to abundance and availability, fewer ethical concerns, low risk of tumorigenicity, and immunomodulatory functions [6].

Adipose tissue‐derived mesenchymal SCs (ASCs) are readily available sources of SCs that can be easily isolated and expanded in vitro, exhibiting an appealing self‐renewal capacity and potential to differentiate into various lineages [7]. Also, ASCs exhibit paracrine activity and secrete cytokines and growth factors such as IL‐1α, IL‐6, and IL‐10, vascular endothelial growth factor (VEGF), fibroblast growth factor (FGF), insulin‐like growth factor (IGF), and platelet‐derived growth factor (PDGF), which are beneficial for cell differentiation and tissue repair [8]. The transplantation of MSCs in patients with bone diseases restored bone structural integrity and functional manifestations without immunologic or neoplastic complications; however, in most cases, low treatment efficacy remained challenging [9–11].

Inducing the osteogenic differentiation of MSCs by manipulating their signaling pathways in vitro before transplantation can improve cell therapy efficacy and enhance bone repair [12]. Autophagy is a fundamental process necessary for degrading and recycling cytoplasmic organelles and components to maintain cellular homeostasis during normal cellular and tissue development [13]. Autophagy is crucial for regulating the self‐renewal and stemness of MSCs [14]. Additionally, autophagy has been shown to promote their osteoblastic commitment, characterized by enhanced expression of *RUNX2*, alkaline phosphatase (*ALP*), osteocalcin (*OCN*), osteopontin (*OPN*), and collagen type I (*COL I*), as well as increased calcium deposition [15].

In vitro osteogenic differentiation is stimulated by treating MSCs with a cocktail of culture media supplemented with small molecules, such as bone morphogenetic proteins (BMPs), dexamethasone, ascorbic acid, and β‐glycerophosphate, which promote osteogenic lineage commitment [16]. Plant‐derived natural compounds have recently garnered increased attention as cost‐effective complementary additives with osteopromotive effects, which involve regulating intracellular osteoblastic signal transduction pathways, such as autophagy [17]. Flavonoids are a class of phenolic compounds found in a variety of fruits, vegetables, seeds, and flowers with pharmacological and biological properties [18].

Apigenin (Api, 4,5,7‐trihydroxyflavone) is a flavonoid phytochemical found abundantly in dietary fruits and vegetables such as celery, parsley, peppers, beans, and chamomile, with extensive antioxidant, anti‐inflammatory, antidiabetic, antitumor, hepatic, neuroprotective, cardioprotective, renoprotective, reproductive, and respiratory protective effects [19]. Several studies have demonstrated that Api can promote the osteogenic differentiation of MSCs by activating signaling pathways, including the Wnt/β‐catenin, JNK, and p38 MAPK pathways [20, 21]. To date, no study has investigated the role of Api in the osteogenic differentiation of ASCs via autophagy induction. In the present study, we evaluated the effect of Api on promoting osteogenic differentiation and stimulating autophagy in ASCs, in vitro.

## 2. Materials and Methods

### 2.1. Isolation and Characterization of ASCs

Fresh adipose tissue samples were obtained from liposuction aspirates of healthy donors (*n* = 2) and transferred to the laboratory. Tissue samples were washed with phosphate‐buffered saline (PBS) containing 2X antibiotics, chopped into tiny pieces, and digested using 2 mg/mL collagenase type I (Cat number SCR103, Sigma–Aldrich Chemie GmbH, Taufkirchen, Germany) under gentle shaking at 37°C for 1 h. Then, tissue digests were centrifuged, and stromal vascular fraction (SVF) was transferred to T25 cell culture flasks containing DMEM‐F12 medium supplemented with 10% fetal bovine serum (FBS, Cat number 16‐000‐044, Gibco Invitrogen, Massachusetts, USA), and 100 units/mL penicillin and 100 µg/mL streptomycin (Cat number 1514022, Gibco Invitrogen, Massachusetts, USA). Flasks were incubated under standard culture conditions at 37°C, 95% humidity, and 5% CO_2_ until isolated ASCs reached 80% confluency. At this time, cells were detached using trypsin/EDTA 0.25%, centrifuged at 2000 rpm for 5 min, and the cell pellet was transferred to two T25 flasks (cell passage). Cells at passage 4 (P4) were selected for the immunocharacterization assay to minimize potential phenotypic drift. The expression of MSC positive markers CD73, CD90, and CD105, and negative markers CD34, and CD45 was assessed by flow cytometry, using antibodies listed in Table 1.

**Table 1:** Antibodies and control isotypes for characterizing ASCs using flow cytometry.

Antibody	Control isotype
PE anti‐human CD73, Clone AD2, Cat # 344003 (Biolegend, USA)	PE Mouse IgG1, κ Isotype, Clone MOPC‐21, Cat # 400111 (Biolegend, USA)
APC anti‐human CD90, Clone 5E10, Cat # 328114 (Biolegend, USA)	APC Mouse IgG1, κ Isotype, Clone MOPC‐21, Cat # 400122 (Biolegend, USA)
PE anti‐human CD105, Clone 43A3, Cat # 323206 (Biolegend, USA)	PE Mouse IgG1, κ Isotype, Clone MOPC‐21, Cat # 400114 (Biolegend, USA)
PE anti‐human CD34, Clone 561, Cat # 343605 ((Biolegend, USA)	PE Mouse IgG2a, κ Isotype, Clone MOPC‐173, Cat # 400211 (Biolegend, USA)
FITC Anti‐CD45 antibody, Clone 2D1, Cat # ab210220 (Abcam, USA)	FITC Mouse IgG1 [B11/6], Cat # ab91356 (Abcam, USA)

### 2.2. Cell Proliferation and Api Dose Determination

To determine the optimum dose of Api without toxicity against ASCs, an MTT assay was performed. P4 ASCs were used for functional assays to ensure high purity and sufficient cell number without compromising stemness. ASCs were seeded into 96‐well plates (P4, 1 × 10^4^ cells/well) and treated with Api (Cat number 10798, Sigma–Aldrich Chemie GmbH, Taufkirchen, Germany) at 0, 5, 10, 25, and 50 µM for 48 and 72 h. At each time, the culture media were removed, and 100 µL of MTT solution (0.5 mg/mL in PBS) (Cat number M6494, Gibco Invitrogen, Massachusetts, USA) was added to each well. The plate was incubated at 37°C in the dark for 3 h, followed by discarding the MTT reagent and adding dimethyl sulfoxide (DMSO, Cat number D8418, Sigma–Aldrich Chemie GmbH, Taufkirchen, Germany). The optical density (OD) of samples was measured at 570 nm using a STAT FAX 2100 microplate reader (Awareness Technology, Inc., Minnesota, USA). Cell proliferation was calculated using Equation (1):

(1) Cell proliferation %= ODTODC ×100,

where OD_*T*_ refers to the absorbance of treatment samples, and OD_*C*_ refers to the absorbance of control samples.

### 2.3. Osteogenic Differentiation

ASCs were cultured in six‐well plates (P4, 3 × 10^5^) and incubated under standard culture conditions as described earlier. Osteogenic differentiation was induced by cultivating cells in DMEM/F12 medium supplemented with 10% FBS, 100 nM dexamethasone (Cat number D4902), 10 nM β‐glycerophosphate (Cat number G9422), and 100 µg/mL ascorbic acid (Cat number A92902) (all from Sigma–Aldrich Chemie GmbH, Taufkirchen, Germany) for 7 and 21 days. Cultivated cells were treated with Api at 0, 5, and 10 µM doses, either as a single dose (on Day 1) or as two doses (on Days 1 and 15), depending on the duration of differentiation. The applied dose–time profiles were selected to coordinate Api exposure with early and late stages of osteogenic differentiation. Cell morphological changes during osteogenic differentiation were observed on Days 7 and 21 using an inverted microscope (Nikon Instruments Inc., USA).

### 2.4. Real‐Time Polymerase Chain Reaction (PCR)

The expression of osteogenic (*ALP*, *RUNX2*, *COL I*, and *OCN*) and autophagic (*ATG5*, *ATG7*, and *LC3A*) genes was evaluated using real‐time PCR on Days 7 and 21. At each time, cells were collected using trypsinization, and total RNA was extracted using the Trizol reagent (Life Biolab GmbH, Heidelberg, Germany) and quantified using a Nanodrop spectrophotometer. cDNA was prepared from RNA using the RevertAid First Strand cDNA Synthesis Kit (Cat number K1622, ThermoFisher Scientific Inc., Porto Salvo, Portugal). Real‐time PCR was performed using cDNA, specific forward and reverse primers, and the high‐ROX RealQ Plus 2x master mix (Cat number A323402, Ampliqon, Odense, Denmark) in the Step One instrument (Applied Biosystems, USA). The threshold cycle (*C*_t_) values were normalized against the housekeeping gene *glyceraldehyde-3-phosphate dehydrogenase (GAPDH)*. Relative gene expression was reported as the fold change = 2^−ddC^ *t*. Forward and reverse primers are listed in Table 2.

**Table 2:** The list of forward and reverse primers used for real‐time PCR.

Gene	Accession number	Sequence (5’→ 3’)	Length (bp)	Temperature (°C)
*ALPL-Human*	NM_001127501.4	F: TGGCAACTCTATCTTTGGTCTGG R: CCGCCCACCACCTTGTAG	23 18	60
*RUNX2-Human*	NM_001015051.4	F: GAACCCAGAAGGCACAGACAG R: GCGGGACACCTACTCTCATAC	21 21	60
*OCN-Human*	NM_199173.6	F: GCAGAGTCCAGCAAAGGTG R: CCAGCCATTGATACAGGTAGC	19 21	60
*COLI-Human*	NM_000088.4	F: TGGAGCAAGAGGCGAGAG R: CACCAGCATCACCCTTAGC	18 19	60
*ATG5-Human*	XM_054356851.1	F: GCAGATGGACAGTTGCACACAC R: GAGGTGTTTCCAACATTGGCTCA	22 22	60
*ATG7-Human*	XM_054344959.1	F: CGTTGCCCACAGCATCATCTTC R: CACTGAGGTTCACCATCCTTGG	22 22	60
*LC3A-Human*	XM_054324147.1	R: CTGGTTCACCAGCAGGAAGAAG F: TGTGTAGCGTCTGCGAGGGAAA	22 22	60
*GAPDH-Human*	NM_001289745.3	F: AAGGTCGGAGTCAACGGATTTG R: GCCATGGGTGGAATCAtATTGG	22 22	60

### 2.5. Western Blot

The production of *RUNX2*, *OCN*, LC3‐I, and LC3‐II proteins was evaluated using Western blot on Days 7 and 21 of osteogenic differentiation. ASCs were trypsinized and centrifuged, and the cell pellet was collected. Then, 1 mL of RIPA lysis buffer was added to the cell pellet, followed by centrifugation at 14,000 rpm (4°C) for 20 min. Next, the supernatant was mixed with an equal volume of 2X Laemmli sample buffer and subsequently boiled in a 100°C water bath for 5 min. Protein samples were quantified using a Bradford assay, diluted in a loading buffer (1:1 ratio) to a final concentration of 1 mg/mL, and subjected to sodium dodecyl sulfate‐polyacrylamide gel electrophoresis (SDS‐PAGE). SDS‐PAGE was run in an electrophoresis tank containing a Tris‐based running buffer at 100 V for 1 h. Next, the protein bands were transferred to 0.45 µm polyvinylidene difluoride (PVDF) transfer paper (Cat number 10600023, Amersham, USA) using the wet transfer method at 60 V for 100 min. The transfer paper was washed three times with PBS and blocked in a 5% skim milk solution at 4°C for 2 h. After washing with PBS, the protein bands were incubated with primary (1:500) and horseradish peroxidase (HRP)‐conjugated secondary (1:1000) antibodies at 25°C for 2 and 1 h, respectively, with washing in PBS at the end of each incubation. After immersing in an enhanced chemiluminescence (ECL) solution, bands were visualized and analyzed using the FUSIONFX ChemiDoc imaging system and software (Vilber, USA), and the protein of interest/β‐actin ratio was reported. The primary and secondary antibodies used for the Western blot are listed in Table 3.

**Table 3:** Primary and secondary antibodies used for the Western blot analysis.

Antibody	Cat #	Company
Anti‐*RUNX2*	TA425912	OriGene Technologies, Inc., USA
Anti‐*OCN*	SL4917R	SunLong Biotech Co., Ltd., China
Anti‐LC3‐I	4599	Cell Signaling Technology, USA
Anti‐LC3‐II	2775	Cell Signaling Technology, USA
Anti‐β‐actin	4970	Cell Signaling Technology, USA
Mouse anti‐rabbit IgG‐HRP	Sc‐2357	Santa Cruz Biotechnology, Inc., USA

### 2.6. *ALP* Activity Assessment

*ALP* activity was evaluated on Days 7 and 21 of osteogenic differentiation. The cultivated cells were washed with PBS and then lysed by adding a 1% Triton X‐100 solution. 250 µL of cell lysate was mixed with an equal volume of p‐nitrophenyl phosphate (p‐NPP, Cat number MBS639297, MyBioSource, Inc., CA, USA) and incubated at room temperature for 1 h. The reaction was stopped by adding 3 M NaOH, and the OD values were read at 405 nm using an ELISA reader. *ALP* activity was calculated against the p‐nitrophenol standard curve.

### 2.7. Quantitative Alizarin Red Staining

Calcium deposition was assessed using Alizarin red staining on Days 7 and 21 of osteogenic differentiation. Cultivated cells were washed with PBS, fixed in 4% paraformaldehyde, and incubated in Alizarin red stain solution (40 mM) for 1 h at room temperature. Then, cells were rinsed in deionized water and incubated with 10% acetic acid for 45 min under gentle shaking. The extracted dye was collected, boiled at 85°C for 10 min, and then cooled to room temperature. The solution pH was adjusted to 4.5 using ammonium chloride, and the ODs were measured at 405 nm. Calcium deposition was determined by comparison with the Alizarin red standard curve.

### 2.8. Data Analysis

All experiments were performed in triplicate (*n* = 3), and data were reported as mean ± SD. The one‐way analysis of variance (ANOVA) followed by Tukey’s test was conducted at the significance level of *p*  < 0.03 using the GraphPad Prism software (version 10). The significance level (*p*  < 0.03) was reported due to the software’s default threshold, and the data were also statistically confirmed under the *p*  < 0.05 criterion.

## 3. Results

### 3.1. ASC Isolation and Characterization

**Figure 1:**
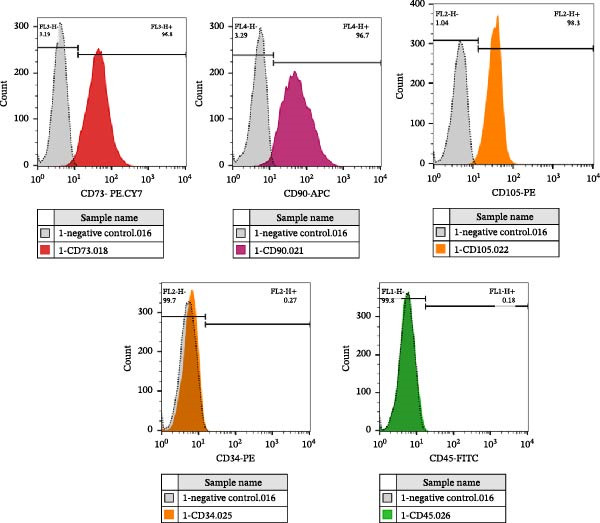
The immunophenotype characterization of ASCs using flow cytometry.

ASCs were isolated, purified, and characterized using flow cytometry (Figure 1). The ASCs expressed CD73 (96.8%), CD90 (96.7%), and CD105 (98.3%), but not CD34 (99.7%) and CD45 (99.8%).

### 3.2. Cell Proliferation and Api Dose Determination

**Figure 2:**
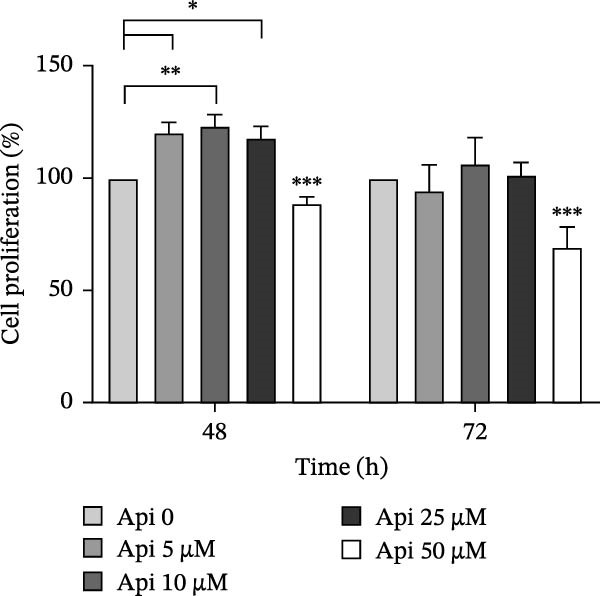
The assessment of the proliferation of ASCs treated with various doses of Api for 48 and 72 h. ASCs, adipose tissue‐derived mesenchymal stem cells; Api, apigenin ( ^∗∗∗^ *p* < 0.001,  ^∗∗^ *p* < 0.002,  ^∗^ *p* < 0.03).

ASCs were treated with Api at various doses for 48 and 72 h, and their proliferation was evaluated using the MTT assay (Figure 2). The results showed that cell proliferation was higher in groups treated with 5, 10, and 25 µM Api by 120.35 ± 4.4%, 123.25 ± 5%, and 117.9 ± 5% after 48 h, and by 94.5 ± 11.3%, 106.4 ± 11.5%, and 101.5 ± 5.4% after 72 h. In contrast, cell proliferation decreased in groups treated with Api 50 µM by 88.7 ± 2.9% and 69.1 ± 9% in 48 and 72 h, respectively ( ^∗∗∗^ *p*  < 0.001,  ^∗∗^ *p*  < 0.002,  ^∗^ *p*  < 0.03). Accordingly, Api doses of 5–10 µM were selected for further experiments.

### 3.3. Cell Morphology Observation

**Figure 3:**
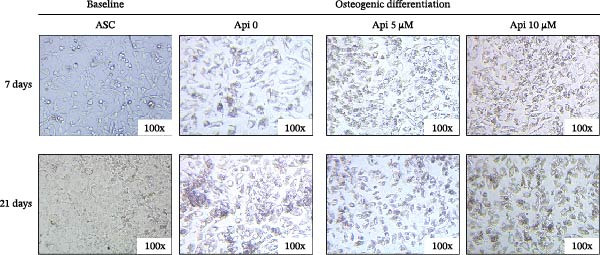
The observational assessment of morphological changes in ASCs differentiated into the osteogenic lineage. The baseline group consists of ASCs cultured in complete medium without undergoing osteogenic differentiation. ASCs, adipose tissue‐derived mesenchymal stem cells; Api, apigenin.

Morphological changes in ASCs undergoing osteogenic differentiation were observed using an inverted microscope on Days 7 and 21 (Figure 3). In the baseline state, when ASCs were cultured in conventional media without osteogenic inducers, they exhibited fibroblast‐like spindle morphology with high proliferation potential. At early stages of osteogenic differentiation (Day 7), cell proliferation decreased, and spindle‐shaped morphology changed toward greater spreading and polygonal morphology. At the terminal stages (Day 21), cells were predominantly polygonal or cuboidal, with thickened cytoplasm, forming clusters resembling osteoblasts. Morphological alteration was more apparent in the Api‐treated groups, where polygonal morphology and cell clusters formed more rapidly and intensively.

### 3.4. Gene Expression Analysis

The expression of osteogenic (*ALP*, *RUNX2*, *COL I*, and *OCN*) and ATG‐related (*ATG5*, *ATG7*, and *LC3A*) genes was evaluated on Days 7 and 21 of osteogenic differentiation (Figure 4). The expression of genes increased significantly on Day 21 compared to Day 7 ( ^∗∗∗^ *p*  < 0.001). *ALP* expression in Api 5 and Api 10 groups was 3.65 ± 0.3 and 4.65 ± 0.85 folds on Day 7 and 12.8 ± 1.7 and 30.5 ± 0.3 folds on Day 21, significantly higher than the control (Api 0) group ( ^∗∗∗^ *p*  < 0.001,  ^∗∗^ *p*  < 0.002,  ^∗^ *p*  < 0.03) (Figure 4a). *RUNX2* levels in the Api 5 and Api 10 groups were 6.15 ± 0.75 and 7.1 ± 1.8 on Day 7, which increased to 11.35 ± 1.35 and 27.7 ± 2.75‐fold on Day 21, with significant differences among groups ( ^∗∗∗^ *p*  < 0.001,  ^∗∗^ *p*  < 0.002, Figure 4b). *COL I* was overexpressed in the Api 5 and Api 10 groups, with values of 5.35 ± 0.9–5.6 ± 1.3 on Day 7, which remarkably elevated on Day 21–7.45 ± 0.6 and 10.7 ± 0.85, respectively ( ^∗∗∗^ *p*  < 0.001, Figure 4c). *OCN* levels in the Api 5 and Api 10 groups were 5.45 ± 0.9 and 7.5 ± 1.25 times higher on Day 7, which peaked at 9.25 ± 1.6 and 15.2 ± 1.1 times higher on Day 21, notably higher than the control group ( ^∗∗∗^ *p*  < 0.001, Figure 4d).

Figure 4The assessment of osteogenic and autophagy gene expression in ASCs treated with Api; (a) *ALP*, (b) *RUNX2*, (c) *COL I*, (d) *OCN*, (e) ATG5, (f) ATG7, and (g) LC3A. *ALP*, alkaline phosphatase; *RUNX2*, runt‐related transcription factor 2; *COL I*, collagen type I; *OCN*, osteocalcin; *ATG5*, *autophagy-related gene 5; ATG7*, *autophagy-related gene 7*; LC3A, microtubule‐associated protein 1 light chain 3A; ASCs, adipose tissue‐derived mesenchymal stem cells; Api, Apigenin ( ^∗∗∗^ *p* < 0.001,  ^∗∗^ *p* < 0.002).

**Figure:**
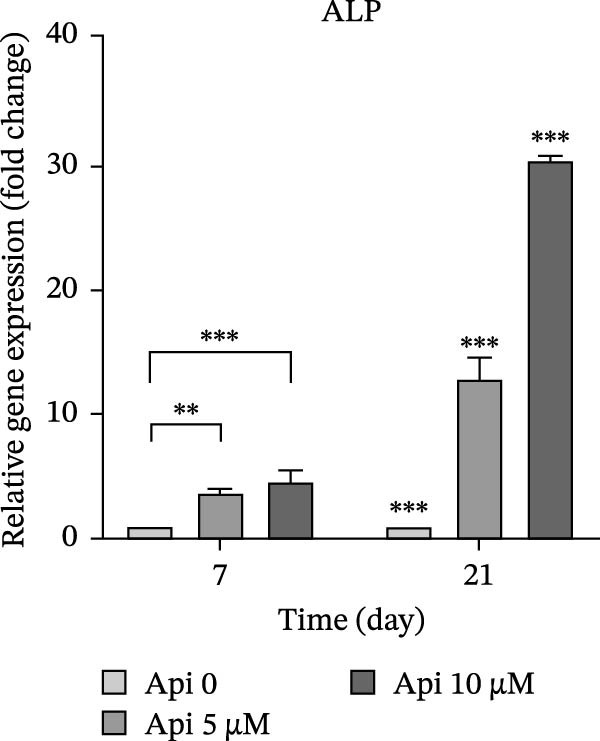
(a)

**Figure:**
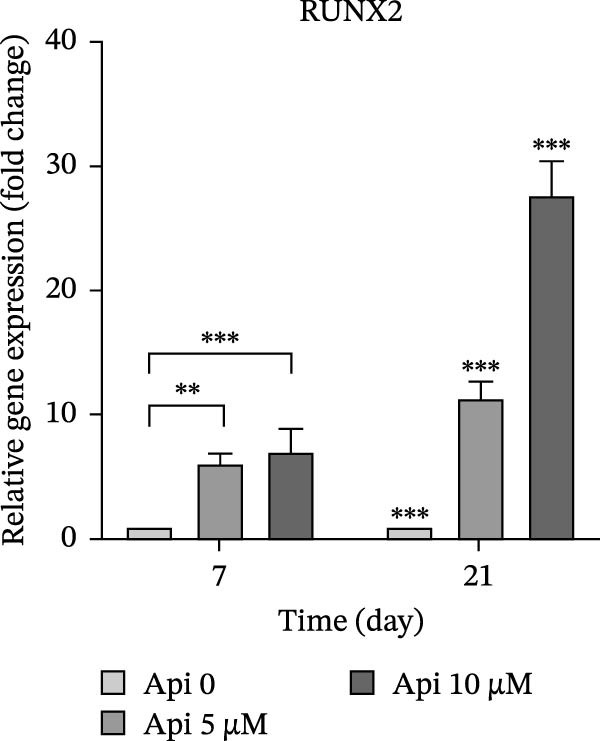
(b)

**Figure:**
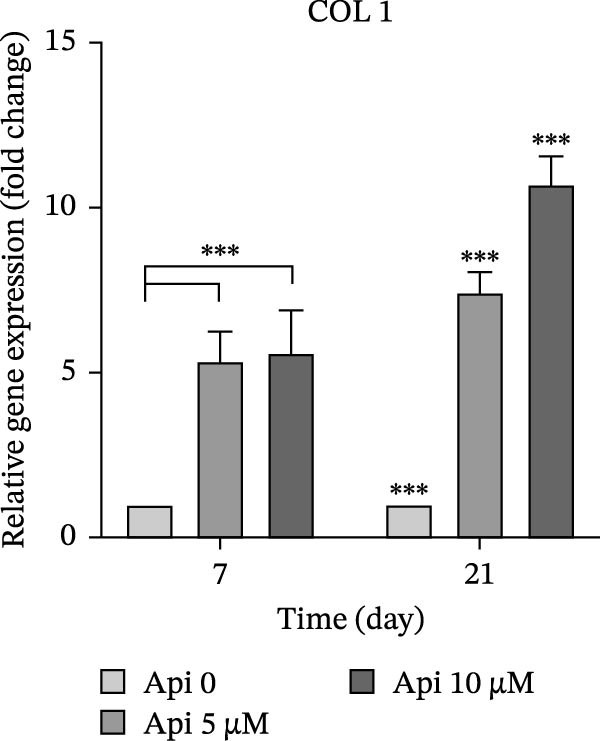
(c)

**Figure:**
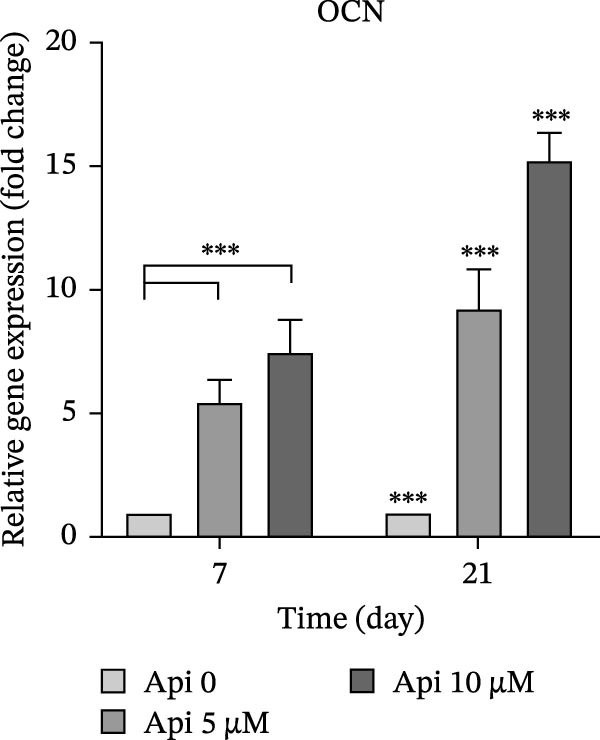
(d)

**Figure:**
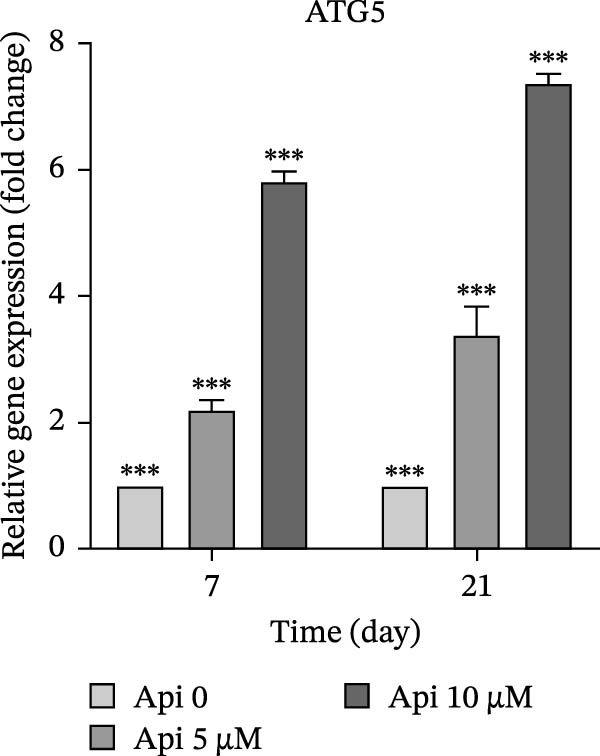
(e)

**Figure:**
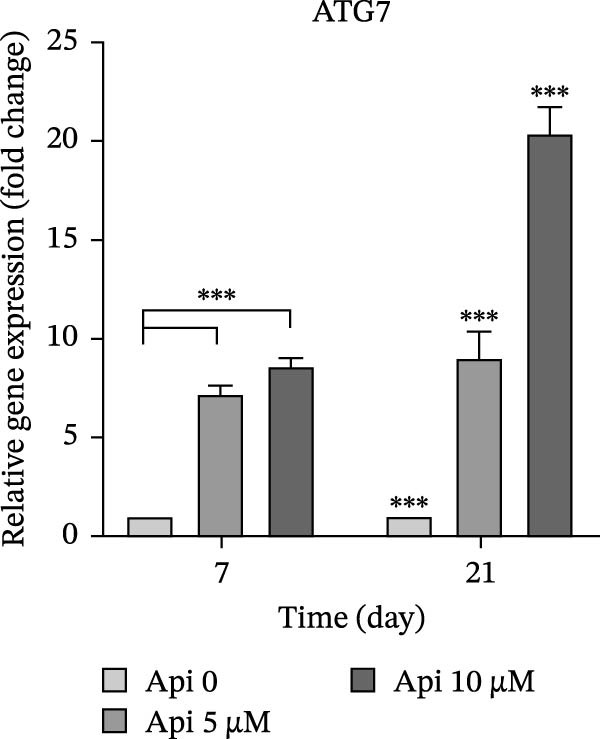
(f)

**Figure:**
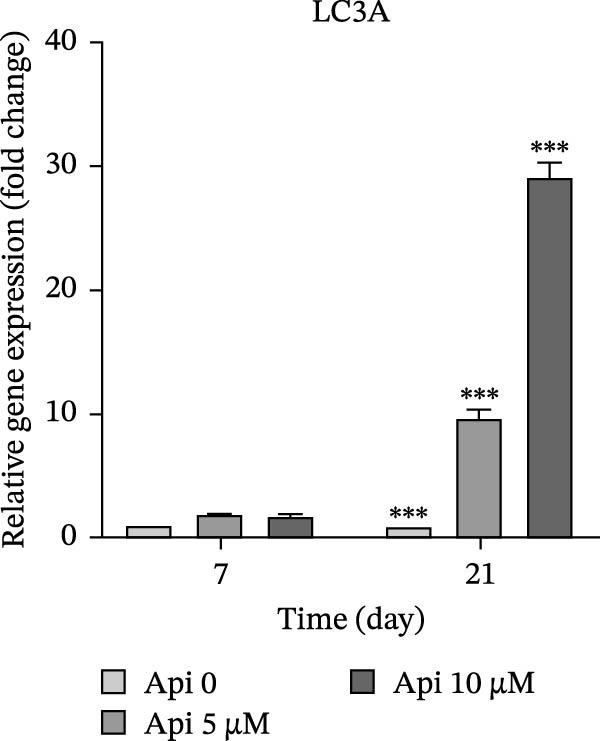
(g)

The expression of ATG5 in Api 5 and Api 10 groups was 2.2 ± 0.15 and 5.8 ± 0.15 folds on Day 7 and 3.4 ± 0.4 and 7.4 ± 0.15 folds on Day 21, significantly greater than the control group ( ^∗∗∗^ *p*  < 0.001, Figure 4e). ATG7 levels were 7.2 ± 0.4 and 8.6 ± 0.35 folds on Day 7 and 9 ± 1.35 and 20.4 ± 1.3 folds on Day 21, remarkably higher than the control group ( ^∗∗∗^ *p*  < 0.001, Figure 4f). LC3A values in Api 5 and Api 10 groups were 1.9 ± 0.05 folds and 1.8 ± 0.2 on Day 7 and 9.4 ± 1 and 29.1 ± 1.2 folds on Day 21, with a notable difference from the control group ( ^∗∗∗^ *p*  < 0.001, Figure 4g).

### 3.5. Protein Production Analysis

The osteogenic (*RUNX2* and *OCN*) and ATG‐related (LC3‐I and LC3‐II) proteins were evaluated on Days 7 and 21 of osteogenic differentiation (Figure 5). The *RUNX2*/β actin ratio in Api 5 and Api 10 groups was 1.25 ± 0.03 and 1.7 ± 0.06 on Day 7 and 8.45 ± 0.05 and 6.6 ± 0.1 on Day 21, with significant differences among groups ( ^∗∗∗^ *p*  < 0.001, Figure 5a). The *OCN*/β actin ratio in Api 5 and Api 10 groups was 0.95 ± 0.1 and 1.75 ± 0.1 on Day 7 and 8.75 ± 0.15 and 10.9 ± 0.1 on Day 21 ( ^∗∗∗^ *p*  < 0.001, Figure 5b). The LC3‐I/β actin ratio in Api 5 and Api 10 groups was 1.3 ± 0.05 and 1.65 ± 0.05 on Day 7, which expanded to 3.7 ± 0.03 and 4.2 ± 0.15 on Day 21, with notable differences from the control group ( ^∗∗∗^ *p*  < 0.001,  ^∗∗^ *p*  < 0.002, Figure 5c). The LC3‐II/ β actin ratio in Api 5 and Api 10 groups was 1.5 ± 0.05 and 1.9 ± 0.05 on Day 7 that raised to 3.3 ± 0.1 and 4.5 ± 0.02 on Day 21, with significant differences ( ^∗∗∗^ *p*  < 0.001, Figure 5d). The LC3‐II/LC3‐I ratio in Api 5 and Api 10 groups was 1.2 ± 0.03 and 1.2 ± 0.02 on Day 7 and 1 ± 0.03 and 1.1 ± 0.02 on Day 21, with a remarkable increase compared to the control group ( ^∗∗∗^ *p*  < 0.001, Figure 5e). The protein bands are shown in Figure 5f.

Figure 5The assessment of the production of osteogenic and autophagy proteins in ASCs treated with Api (a) *RUNX2*, (b) *OCN*, (c)LC3‐I, (d) LC3‐II, (e) LC3‐II/LC3‐I ratio, and (f) protein bands detected using Western blot. ASCs, adipose tissue‐derived mesenchymal stem cells; *RUNX2*, runt‐related transcription factor 2; *OCN*, osteocalcin; LC3, microtubule‐associated protein 1 light chain 3; Api, Apigenin ( ^∗∗∗^ *p* < 0.001,  ^∗∗^ *p* < 0.002).

**Figure:**
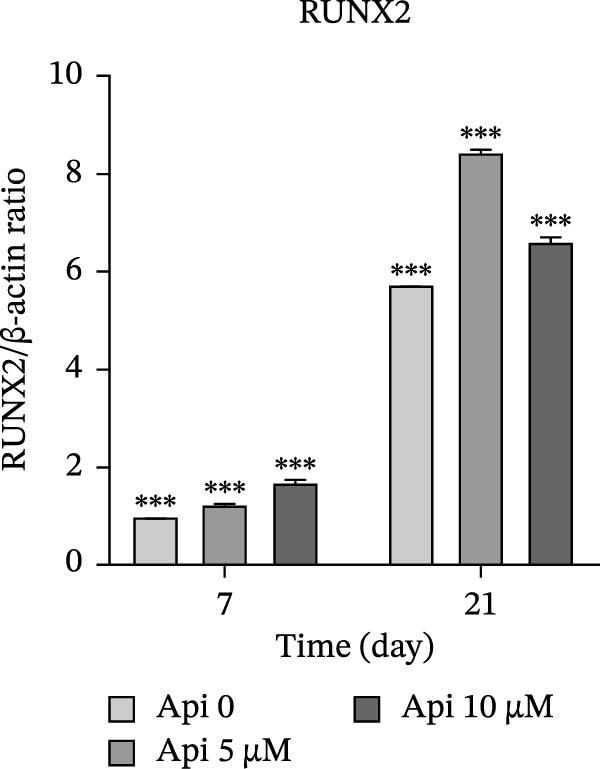
(a)

**Figure:**
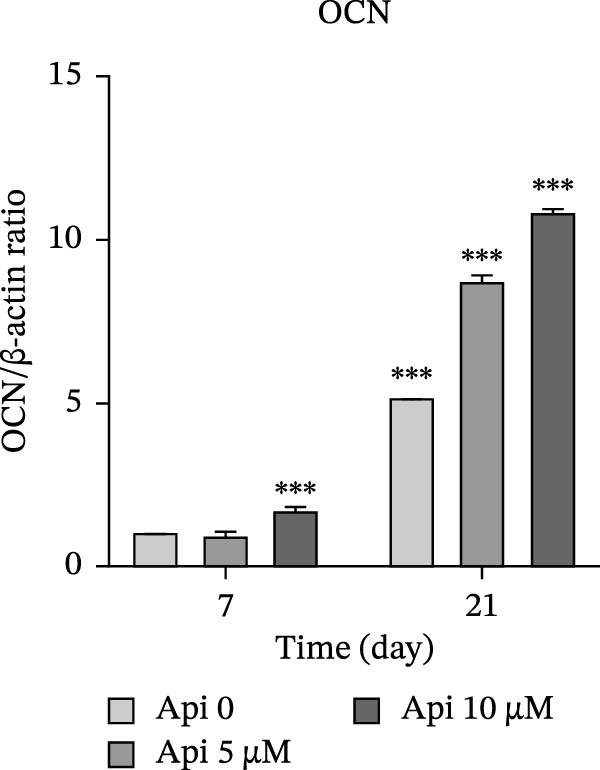
(b)

**Figure:**
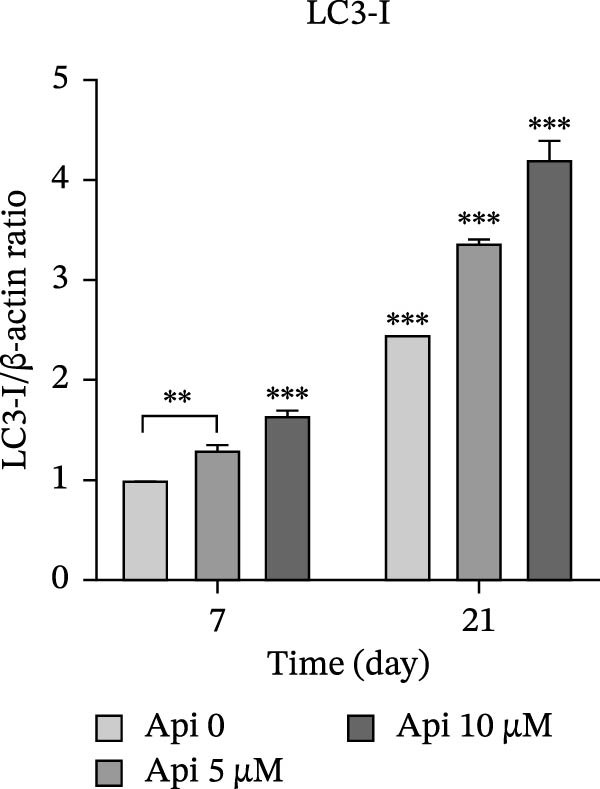
(c)

**Figure:**
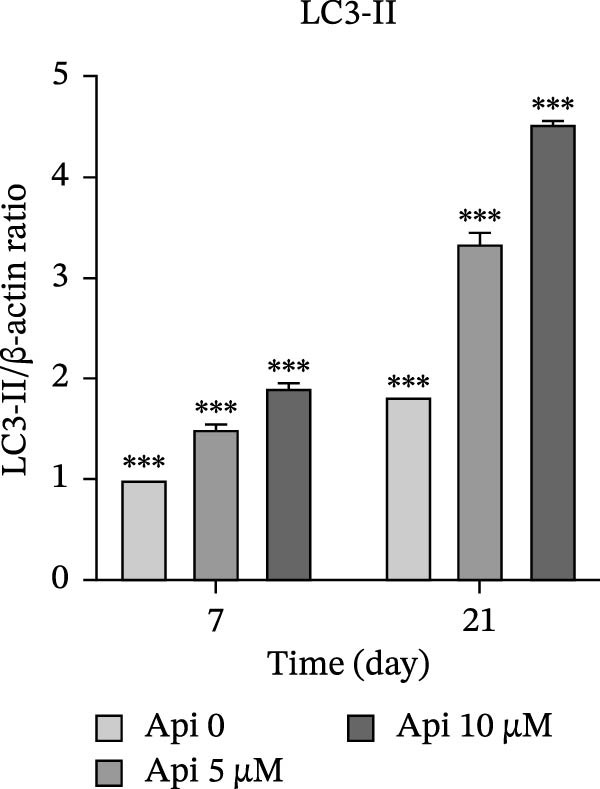
(d)

**Figure:**
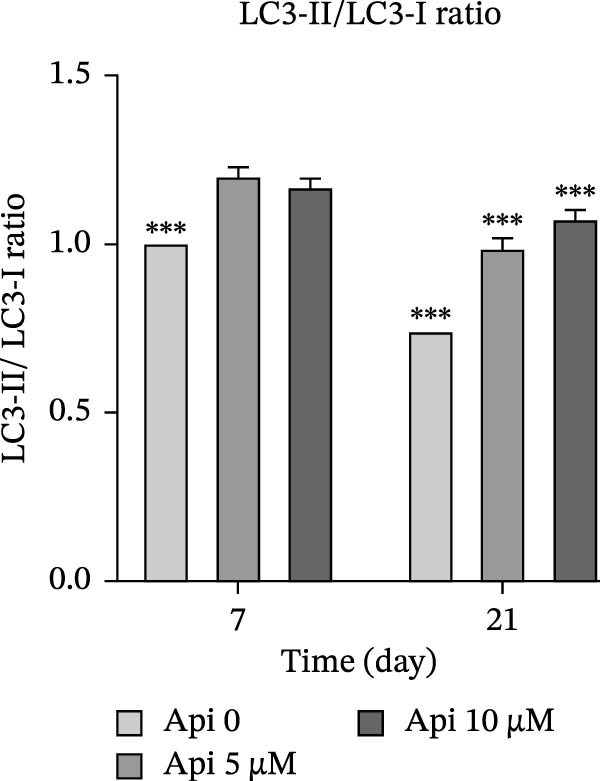
(e)

**Figure:**
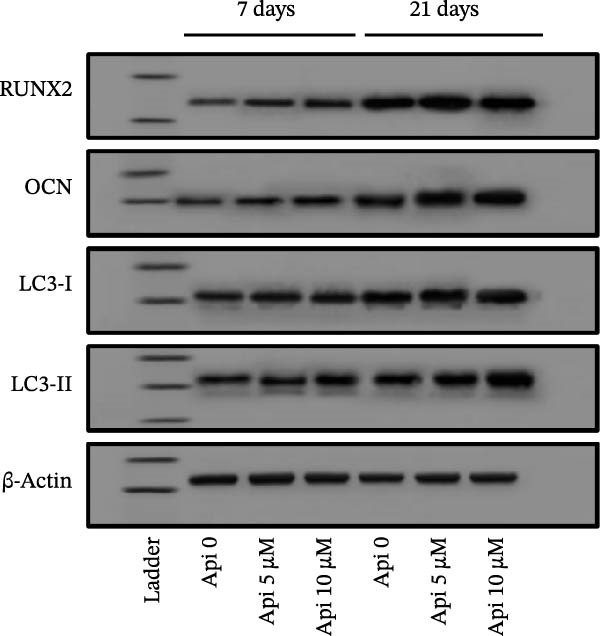
(f)

### 3.6. *ALP* Activity

*ALP* activity was evaluated on Days 7 and 21 of the osteogenic differentiation (Figure 6a). *ALP* activity escalated in all groups from Days 7 to 21 ( ^∗∗∗^ *p* < 0.001). *ALP* activity in Api 0, Api 5, and Api 10 groups was 0.83 ± 0.01, 0.85 ± 0.15, and 0.9 ± 0.01 units/µl of total protein on Day 7 and 1.15 ± 0.03, 1.15 ± 0.01, and 1.2 ± 0.04 units/µl of total protein on Day 21. *ALP* activity was higher in groups treated with Api, but the significant difference was only observed between the control and Api 10 µM group ( ^∗^ *p* < 0.05).

Figure 6The assessment of *ALP* activity and calcium deposition in ASCs treated with Api; (a) *ALP* activity, (b) Alizarin red S staining. *ALP*, alkaline phosphatase; ASCs, adipose tissue‐derived mesenchymal stem cells; Api, apigenin ( ^∗∗∗^ *p* < 0.001,  ^∗∗^ *p* < 0.002,  ^∗^ *p* < 0.03).

**Figure:**
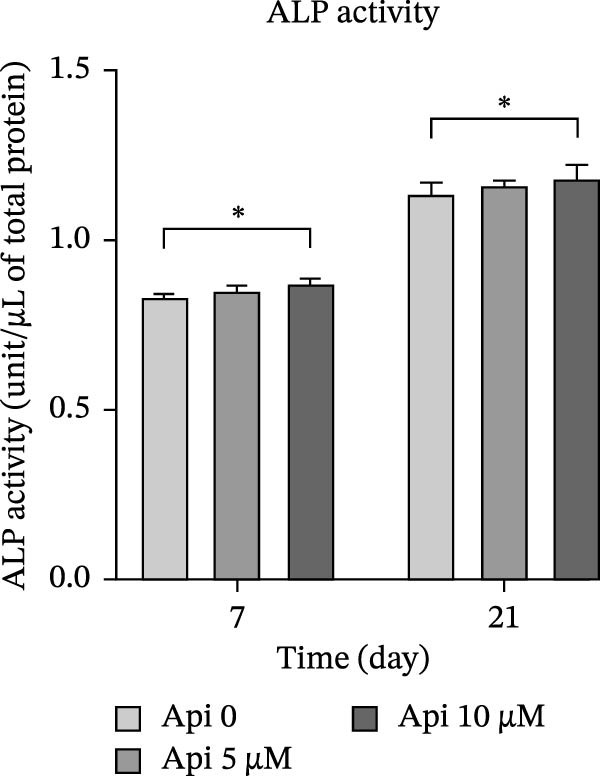
(a)

**Figure:**
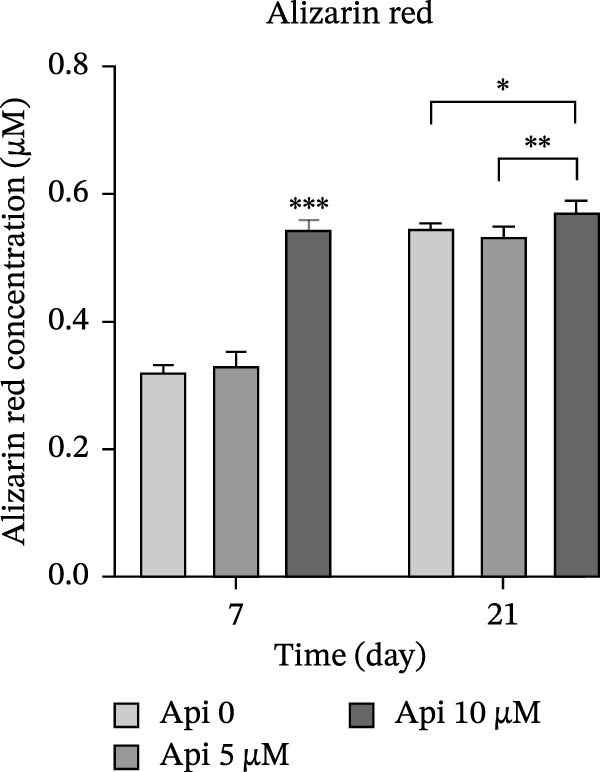
(b)

### 3.7. Quantitative Alizarin Red S Staining

Calcium deposition was evaluated on Days 7 and 21 of osteogenic differentiation using a quantitative Alizarin Red S staining method (Figure 6b). Results showed that cellular mineralization increased in all groups in a time‐dependent manner ( ^∗∗∗^ *p*  < 0.001). Alizarin red concentration in Api 0, Api 5, and Api 10 groups was 0.32 ± 0.01, 0.33 ± 0.02, and 0.55 ± 0.01 µM on Day 7, rising to 0.55 ± 0.01, 0.535 ± 0.15, and 0.6 ± 0.02 µM on Day 21, with significantly higher levels in the Api 10 µM group ( ^∗∗∗^ *p* < 0.001,  ^∗∗^ *p* < 0.01,  ^∗^ *p* < 0.05).

## 4. Discussion

In the present work, we evaluated the effect of Api on the osteogenic differentiation of ASCs via inducing autophagy. Bone remodeling is a dynamic process in which the coordinated function of osteoblasts and osteoclasts maintains the integrity of the bone matrix [22]. Despite the innate regenerative potential of bone, challenging critical bone defects that occur following inborn deformations, traumas, fractures, and accidents require extrinsic therapeutic interventions [23]. SC therapy and regenerative medicine offer a promising approach for managing and treating challenging bone defects that are difficult to address with conventional methods. MSCs are an affordable source of SCs found in postnatal and adult tissues, offering easy access and a high yield, and can be expanded in vitro and differentiated into various cell types, including osteoblasts [24]. Additionally, MSCs act as reservoir progenitors participating in tissue homeostasis and regeneration while secreting cytokines and growth factors with anti‐inflammatory, angiogenic, regenerative, and immunomodulatory activities [25]. Using osteogenesis‐inducing phytochemicals has been highlighted for enhancing differentiation efficiency through activating cellular signaling pathways [26].

In the current experiment, MSCs were isolated from adipose tissue and characterized using flow cytometry. Isolated ASCs were highly pure, with over 94% of cells displaying the conventional MSC immunophenotype (CD73^+^, CD90^+^, and CD105^+^), and were negative for CD34 and CD45. Our findings indicated that Api at 5 and 10 µM had no toxicity to ASCs, whereas higher doses reduced cell proliferation. This data was consistent with a previous study showing that Api at doses of 2.5–20 µM increased natural killer (NK) cell proliferation. Additionally, 5 and 10 µM doses of Api substantially upregulated the antiapoptotic BCL‐2 while downregulating the proapoptotic BAX [27].

Our results showed that Api overexpressed *ALP*, *RUNX2*, *OCN*, and *COL I* and exhibited higher *ALP* activity and calcium deposition in a dose‐dependent manner. *ALP* is an ectoenzyme anchored to the glycosyl phosphatidyl inositol and a transient representative of osteoblastic differentiation that catalyzes phosphate esters into inorganic phosphorus and promotes matrix mineralization [28]. *RUNX2* is a main transcription factor of osteogenesis that regulates the expression of osteogenesis‐related genes, bone sialoprotein (*BPN*), *OPN*, *COL I*, and *OCN* in differentiating MSCs [29]. *RUNX2* protein levels decreased on Day 21 in all experimental groups compared to Day 7, reflecting posttranslational mechanisms that drive *RUNX2* protein degradation and ensure proper osteogenic differentiation and osteoblast maturation [30].

*COL I* is the main substance protein found in bone matrix, providing a framework for osteoblast mineralization [31]. *OCN* is the most abundant noncollagenous protein in bone matrix that binds Ca ions with high affinity, thereby enhancing matrix mineralization [32]. In this article, the stronger *ALP* activity, accompanied by increased *OCN* levels, in Api‐treated cells led to higher mineralization rates. Matrix mineralization is a process in which hydroxyapatite crystals are deposited throughout the protein substrate and serve as a biological marker for assessing the osteoblastic commitment of MSCs [33].

Previous investigations have shown that Api promotes osteogenic differentiation of MSCs through multiple mechanisms. Zhang et al. [21] demonstrated that Api at 0.1, 1, and 5 µM induced MSCs to adopt an osteogenic lineage via activation of the JNK/p38 MAPK signaling pathway, resulting in increased *ALP*, *RUNX2*, and OPN. Api augmented osteoblastic differentiation via intensifying the β‐catenin and downstream target elements in the Wnt signaling cascade, resulting in accelerated bone healing in vivo [20]. Additionally, local treatment with Api improved the osteogenic commitment of human dental pulp SCs (hDPSCs) by modulating inflammatory cytokines, including TNF‐α, TGF‐β1, and myeloperoxidase (MPO), enhancing Nestin expression, and ultimately enhancing tertiary dentin repair [34].

Additionally, this work revealed that Api increased the expression of autophagy‐related genes *ATG5*, *ATG*, and *LC3A*, as well as the production of LC3‐I and LC3‐II proteins. ATG is essential for regulating the osteogenic differentiation of MSCs by modulating cellular signaling pathways and maintaining cellular homeostasis. This highly conserved catabolic process is orchestrated by autophagy‐related elements that actively degrade damaged organelles and unnecessary macromolecules, then recycling them for use in cell metabolism [35]. ATG5 is a key component for autophagosome formation and expansion, forming a complex with ATG12 to facilitate LC3‐I conversion [36]. Zhao et al. [37] exhibited that ATG5 was essential for BMP‐9‐induced osteogenic differentiation of MSCs. Silencing ATG5 in MSCs effectively reduced BMP9 signaling activity and suppressed *RUNX2* and OPN expression [37]. ATG7 is an E1‐like ubiquitin‐activating enzyme that catalyzes the conjugation of ATG12–ATG5 and the conversion of LC3‐I–LC3‐II.

LC3‐I is the cytosolic form of LC3, while LC3‐II is the autophagosome‐associated form, where the LC3‐II/LC3‐I ratio indicates autophagosome formation and autophagy progression [38]. Our findings showed that the expression of autophagic genes increased during differentiation; however, the LC3‐II/LC3‐I ratio declined by Day 21 compared to Day 7. A rationale is that autophagy is upregulated in the early stages of differentiation, reflecting the higher metabolic activity of MSCs undergoing differentiation [39]. As differentiation proceeds, MSCs transition from an SC phenotype to osteogenically committed cells, which ultimately form mature osteoblasts with reduced metabolism and lower autophagy flux.

Vidoni et al. [40] reported that resveratrol exerted a synergistic effect on the osteogenic differentiation of human gingiva‐derived MSCs, associated with activation of AMPK/Beclin 1 signaling and autophagosome formation. Although no study has investigated the autophagy‐inducing effect of Api on osteogenic differentiation, some evidence suggests that the activation of the JNK/p38 MAPK pathway in MSCs differentiated into osteoblasts leads to the upregulation of FOXO3, a key transcription factor for autophagy initiation. Consistently, inhibiting ATG7 was linked to impaired osteogenic differentiation [41]. MAPK activation can mediate autophagy‐stimulated osteoblastic commitment of DPSCs via inhibiting mTOR signaling [42]. Additionally, the anti‐inflammatory properties of Api may be effective in inducing autophagy, as demonstrated by Gao et al. [43], who showed that inhibiting the NF‐κB signaling pathway led to JNK activation and autophagy induction in porcine granulosa cells, thereby enhancing their secretory function.

## 5. Conclusion

In summary, this article was an exploratory study that provided preliminary insights into the role of Api in improving the osteogenic differentiation of ASCs by stimulating autophagy. Considering phytochemicals and the mechanisms by which they promote the osteoblastic differentiation of MSCs is a step forward in developing novel SC‐based strategies for treating bone diseases. However, additional in vitro and in vivo preclinical studies are necessary to verify the safety and efficacy of these strategies before they are translated from bench to bedside. There are some limitations with the current experiment that should be considered for further studies. The sample size and the number of adipose tissues obtained from donors were too small, which might limit the generalizability of the findings.

## Author Contributions

Mozafar Khazaei investigated the experiment, performed formal analysis, and wrote and reviewed the original draft. Amirmohammad Khodaei investigated the experiment and reviewed the draft. Maryam Bozorgi investigated the experiment, performed a formal analysis, and reviewed the draft. Mohammad Rasool Khazaei wrote and reviewed the original draft. Azam Bozorgi conceptualized and investigated the experiment, administered the project, performed formal analysis, and wrote the original draft.

## Funding

The present work was financially supported by the Kermanshah University of Medical Sciences, Kermanshah, Iran (Grant 4030127).

## Ethics Statement

The current in vitro experiment was performed according to the guidelines of the Code of Ethics of the World Medical Association (Declaration of Helsinki) principles and approved by the Ethics Committee of Kermanshah University of Medical Sciences, Kermanshah, Iran (Ethics code IR.KUMS.MED.REC.1403.032).

## Conflicts of Interest

The authors declare no conflicts of interest.

## Data Availability

The data that support the findings of this study are available from the corresponding author upon reasonable request.
